# Biocontrol Activity of Aromatic and Medicinal Plants and Their Bioactive Components against Soil-Borne Pathogens

**DOI:** 10.3390/plants12040706

**Published:** 2023-02-05

**Authors:** Babett Greff, András Sáhó, Erika Lakatos, László Varga

**Affiliations:** 1 Department of Food Science, Albert Casimir Faculty at Mosonmagyarovar, Szechenyi Istvan University, 15-17 Lucsony Street, 9200 Mosonmagyarovar, Hungary; 2 Wittmann Antal Multidisciplinary Doctoral School in Plant, Animal, and Food Sciences, Szechenyi Istvan University, 2 Var Square, 9200 Mosonmagyarovar, Hungary; 3 Kisalfoldi Agricultural Ltd., 1 Fo Street, 9072 Nagyszentjanos, Hungary

**Keywords:** biopesticide, aromatic plant, herb, soil-borne pathogen, essential oil

## Abstract

Soil-borne phytopathogens can have detrimental effects on both cereal and horticultural crops resulting in serious losses worldwide. Due to their high efficiency and easy applicability, synthetic pesticides are still the primary choice in modern plant disease control systems, but stringent regulations and increasing environmental concerns make the search for sustainable alternatives more pressing than ever. In addition to the incorporation of botanicals into agricultural practices, the diversification of cropping systems with aromatic and medicinal plants is also an effective tool to control plant diseases through providing nutrients and shaping soil microbial communities. However, these techniques are not universally accepted and may negatively affect soil fertility if their application is not thoroughly controlled. Because the biocontrol potential of aromatic and medicinal plants has been extensively examined over the past decades, the present study aims to overview the recent literature concerning the biopesticide effect of secondary metabolites derived from aromatic and medicinal plants on important soil-borne plant pathogens including bacteria, fungi, and nematodes. Most of the investigated herbs belong to the family of Lamiaceae (e.g., *Origanum* spp., *Salvia* spp., *Thymus* spp., *Mentha* spp., etc.) and have been associated with potent antimicrobial activity, primarily due to their chemical constituents. The most frequently tested organisms include fungi, such as *Rhizoctonia* spp., *Fusarium* spp., and *Phytophthora* spp., which may be highly persistent in soil. Despite the intense research efforts dedicated to the development of plant-based pesticides, only a few species of aromatic herbs are utilized for the production of commercial formulations due to inconsistent efficiency, lack of field verification, costs, and prolonged authorization requirements. However, recycling the wastes from aromatic and medicinal plant-utilizing industries may offer an economically feasible way to improve soil health and reduce environmental burdens at the same time. Overall, this review provides comprehensive knowledge on the efficiency of aromatic herb-based plant protection techniques, and it also highlights the importance of exploiting the residues generated by aromatic plant-utilizing sectors as part of agro-industrial processes.

## 1. Introduction

Continuously increasing human population, ever-growing demand for food, and agricultural intensification have been placing a tremendous pressure on soil health, leading to degradation and exhaustion of agricultural lands and declining yields and productivity [1,2,3,4]. The evaluation of soil health and quality is a critically important step in maintaining long-term agricultural sustainability [5,6]. Healthy soil is characterized by stability, continuous nutrient cycling, stress resilience, richness, evenness, abundance, and diversity of microbial communities [7,8]. The interactions between plants and soil-dwelling microorganisms are important drivers of the agroecosystem, as these indigenous microbes can control the development, defense, tolerance, and nutrition of plants [9,10,11]. However, the composition and abundance of microbial communities are highly influenced by various parameters such as the type of cultivated plants, environmental fluctuations, and conducted agricultural activities [7,10,12].

Reduced plant productivity and phytopathogen-related disease outbreaks can be considered as indicators of soil instability, poor ecosystem health, and a lack of microbial diversity [7,8]. Soil-borne pathogens including viruses, bacteria, fungi, protozoa, and nematodes are well known for their socio-economic and ecological impacts on the ecosystem by causing serious losses in the agro-industry [13,14,15,16]. Plant roots and their exudates provide substrates and space for these phytopathogens leading to colonization and infection. Generally, the eradication of these organisms is especially hard in continuous-cropping systems, due to the intensive tillage and cultivation processes [7,17].

Both agricultural practices and directed agronomical measures can affect the diversity of soil microbial communities [18]. Traditionally, crop rotation techniques had been used by farmers to manage the productivity of soil and reduce the possibility of crop diseases [19]; however, after World War II, shortened rotation periods, intensive tillage, monocropping, and application of synthetic pesticides and fertilizers came to the fore [20]. Although numerous cultural, physical, biological, and genetic options are available nowadays, the main strategies for controlling soil-borne pathogens are still highly dependent on the use of agrochemicals [15,21] due to their high efficiency and easy applicability [22]. Besides the positive effects, the indiscriminate use of these chemical agents may induce undesirable changes to the beneficial soil microorganisms and human and animal health [22,23,24]. The commonly applied chemical pesticides are usually effective against their target organisms, but they are not specific for them [25]. According to Ashraf and Zuhaib [26], only an estimated 0.1% of the used synthetic chemicals reach the targeted pathogens, whereas the remaining 99.9% may contaminate the environment and endanger nontarget organisms.

The application of many chemical agents has been restricted in certain parts of the world [27]. Therefore, finding sustainable and safer solutions with minimum or no side effects is essential to develop effective soil health management strategies and lower the possible environmental pollution [23,28,29,30].

**Figure 1:**
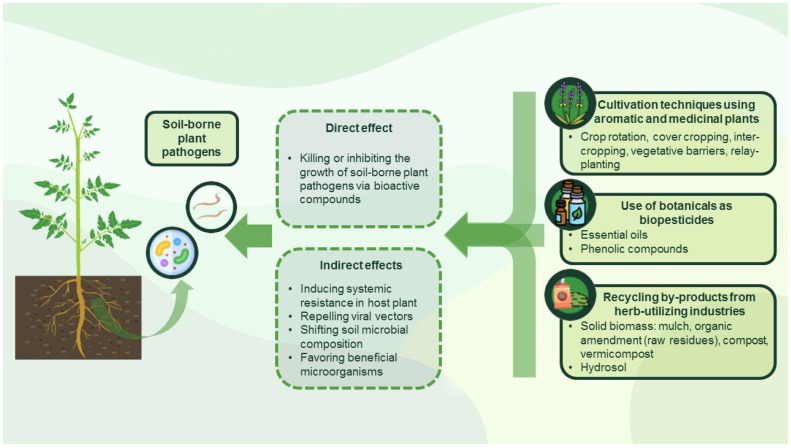
Aromatic and medicinal plants as part of non-chemical plant protections systems: effects and methods of application. The icons in circles were made by Blackwoods, Freepik, Kerismaker, and Surang (www.flaticon.com). The plant illustration on the left is based on the work of Mariya Kazakova (purchased from www.shutterstock.com).

In recent years, there has been an increasing interest in the application of allelochemicals isolated from higher plants in plant protection because they are safer than their synthetic counterparts, effective at reduced doses, and biodegradable without leaving harmful residues [31,32,33,34]. The use of pathogen-suppressing secondary metabolites (e.g., alkaloids, terpenoids, phenols) is especially important in organic farming systems [33,35], but conventional cultivation methods and agricultural practices utilizing aromatic plants and their residues (i.e., solid biomass and hydrosols) may also be effective in controlling the spread and severity of plant diseases (Figure 1).

This paper summarizes and reviews (a) the most important soil-borne pathogens including bacteria, fungi, nematodes, and viruses; (b) the current status of botanical pesticides; and (c) the beneficial effects of aromatic and medicinal plant-based cultivation techniques on soil and plant health. Moreover, the reutilization of by-products generated by aromatic plant-based industries as biocontrol agents is also discussed.

## 2. Phytopathogens in Soil

Soil biology is in direct connection with agricultural sustainability [36], as healthy soil can recycle nutrients, decompose organic matter, support biological activities, suppress the growth of pathogens, and inactivate toxic compounds [37]. It is the habitat of a myriad of organisms (e.g., invertebrate animals, plants, protozoa, fungi, and bacteria) that play an important role in maintaining agro-ecosystem functions and productivity [38,39]. As for microbial diversity, only a few grams of soil can contain millions of species [40]. In addition to beneficial microbes, however, major and minor pathogens may also be present [15,41]. Even though the occurrence of microbial plant diseases is a natural part of crop production, the excessive accumulation and epidemic spread of phytopathogens can induce serious crop damages and yield losses [12,36].

Soil-borne pathogenic fungi, bacteria, protozoa, viruses, and plant parasitic nematodes can cause negative plant–soil feedback individually or collectively [42,43]. They usually affect the root system or the stem base of plants, but vascular diseases caused by these organisms have also been reported [44]. Overall, approximately 15% of total crop production is estimated to be lost to different biological threats annually [45], but microbe-induced diseases may even lead to total crop failure [46].

### 2.1. Fungi

As far as fungal pathogens are concerned, infections can be transmitted through soil-damaging agricultural and horticultural products [47]. *Fusarium*, *Verticillium*, *Rhizoctonia*, *Sclerotinia*, *Phytophthora*, and *Pythium* species may survive in the soil and in plant residues for longer periods by forming resistant structures such as sclerotia, microsclerotia, oospores, chlamydospores, or hyphae [22,48]. Several of them can invade the host plant through roots and stems or spread rapidly among the seedlings [49] causing root necrosis and vascular diseases, as well as rot, gall, and proliferation of roots and tubers [44]. Recent studies have highlighted that the causal agents of root rot (*Fusarium solani* and *Fusarium oxysporum* f. sp. *dahliae-lycopersici*) and wilting (*Verticillium dahliae*) are able to infect a considerable range of crop plants, making the controlling process extremely difficult without high-cost fumigants [50]. For instance, *Verticillium dahliae* can survive in the soil for up to 14 years by forming microsclerotia and may affect more than 160 plant species [51]. Furthermore, fungi belonging to the genera *Pythium* and *Phytophthora* may cause similar symptoms in the crown, stem, and root tissues of certain crops leading to scars; girdled stem; stunt; stem lesions; foliar blight; browning; wilting; decay; damp-off; loss of root density; and rot of crown, roots, and fruits [52,53].

### 2.2. Bacteria

Along with fungal strains, important bacterial pathogens can be found in the soil including *Agrobacterium*, *Pectobacterium*, *Pseudomonas*, *Ralstonia*, and *Xanthomonas* species [36,54]; however, bacterial pathogens are less likely to cause plant diseases, as they need a wound or a natural opening to invade the host plant [16]. *Ralstonia solanacearum* is a causal agent of bacterial wilt disease in various crops, including potato, banana, tomato, and peanut. The bacteria enter the plant through wounds, cracks, or root tips; trigger morphological alterations in the root system of the infected host; invade the xylem vessels; and cause wilting symptoms and plant death [54,55]. *Pseudomonas syringae* and *Pectobacterium carotovorum* are also important soil-borne bacteria inducing necrotic lesions on tomato and carrot soft root, respectively [56]. *Pectobacterium atrosepticum* is mainly responsible for various field symptoms of potato, including reduced emergence, wilting, chlorosis, stem and tuber rot, haulm desiccation, blackleg, and plant death [57]. *Xanthomonas campestris* pv. *musacearum* may infect banana plants through injured roots and stems. Since no remedy is available against this pathogen, the infected plant may be cut down, and fallowing or prolonged crop rotation technique may be introduced [58].

### 2.3. Nematodes

So far, over 4100 species of plant-parasitic nematodes have been identified. They cause serious economic losses globally (approximately USD 173 billion a year [59]) due to their wide variety of interactions and host range. These small roundworms can be categorized as ectoparasitic and endoparasitic species [59,60,61,62,63,64,65,66]. Among them, the sedentary endoparasitic nematodes [66] are the most economically important species. The temperate *Meloidogyne hapla* and the tropical *Meloidogyne incognita, Meloidogyne arenaria*, and *Meloidogyne javanica* belong to the most destructive root-knot nematodes that can infect almost all vascular plant species [61]. The parasitism of these biotrophic organisms includes the establishment of permanent feeding sites in the root cortex, vascular parenchyma, endodermis, and pericycle of host plants. The intensified metabolic activity in the multinucleate giant cells mobilizes photosynthates and, consequently, decreases plant quality and quantity with typical symptoms such as suppressed plant growth, wilting, leaf discoloration, and root deformation [61,67]. Southern knot-nematodes may also interact with soil-borne fungi resulting in disease complexes [62]. Regarding cyst nematodes, potato, cereal, and soybean cyst nematodes have been identified as the most significant obligate biotrophs that may be highly persistent in the soil and can survive for longer periods as eggs inside a cyst without a host making their eradication almost impossible [61,68]. In addition, ectoparasitic nematodes are an integral part of the soil fauna [69] moving freely between root-feeding sites. However, they are more likely exposed to various environmental conditions and predators. Similarly to endoparasites, they cause localized damage weakening the host plant defense against bacterial and fungal infections [70], whereas certain species, including *Xiphinema index*, may act as a vector of viruses [63]. Currently, various methods (e.g., use of chemical agents, bio-fumigants, sanitation, resistant species, solarization, steaming, tilling, fallowing, escape cropping, cover cropping with trap crops, crop rotation with poor or non-hosts, soil amendments, biological control by natural antagonists, etc.) are employed to reduce nematode infestation in the soil [71]. Although chemical nematicides are efficient for the control of root-knot nematodes, their use has been gradually decreased due to their toxicity, limited availability in developing countries, high costs, and impaired efficacy after repeated applications [72,73].

### 2.4. Viruses

Soil may contain enormous amounts of soil-borne plant viruses with an estimated viral abundance of 10^31^ [74]. The annual crop losses caused by these tiny organisms are estimated at USD 60 billion worldwide. Currently, more than 2000 species have been identified that affect economically important crops including barley, groundnut, wheat, sugar beet, potato, and fruit crops [42,75]. The first report was released in 1925 in connection with a soil-borne virus, the soil-borne wheat mosaic virus, and further 53 plant pathogens belonging to 12 genera have been recognized as soil-borne viruses in the last decades [27]. They are generally transmitted via soil, fungi, nematodes, plasmodiophora, insects and other arthropods, sap transmission, seed and pollen transmission, mechanical friction, and vegetative propagation materials [42,74]. In general, they are extremely persistent [42] just as with beet soil-borne mosaic virus that is spread by a soil-borne plasmodiophoromycete, *Polymyxa betae*, which generates resting spores allowing the virus to survive in the soil for decades [76]. Although different viruses have different host ranges, collectively, they can infect almost all kinds of cultivated and noncultivated plants, and the infections can be transmitted from the affected plants to the healthy ones [75]. As an example, the tobacco mosaic virus is a common viral disease that affects over 1000 species in 85 plant families [75,77]. The remains of infected plants in the soil are the principal reservoirs of the virus. Transmission mostly occurs when leaves are rubbed in the presence of virus-containing soil or when injured root hairs come into contact with infected residues or free virus [78]. Furthermore, tomato spotted wilt virus is capable of infecting more than 1000 plant species (e.g., ornamental plants, lettuce, pepper, potato, etc.) [79]. Since the limitation of viral infections and the eradication of viruses from infected soil are almost impossible, control methods have mostly relied on various agrochemicals that can reduce the vector population, resistant plant varieties, and other preventive measures [27,42,80].

## 3. Non-Chemical Plant Protection Methods

The lack of effective pest control is still a major global obstacle to improved plant health and productivity [81]. Synthetic agrochemicals have been widely used to control soil-borne pathogens [21], but the regulations on the application of these products have become more stringent because of their adverse effects [82]. After the implementation of the Montreal Protocol, EU regulations EC 2037/2000 and EC 3093/1994 completely prohibited the use of methyl bromide, the most popular fumigant against soil-borne bacteria, fungi, and nematodes. Furthermore, European Directive 2009/128/EC on the sustainable use of pesticides was issued to reduce the risk of pesticides to the environment and human health [22,28,83,84,85], and European Regulation no. 1107/2009 has been controlling the placement of plant protection products on the market [86]. This led to the prohibition of several traditional pesticides [87] and fueled the search for other suitable candidates, including existing and new agrochemicals and more sustainable, economically feasible alternatives that are less dangerous to the environment [88,89]. However, these methods must meet various standards regarding pest specificity, toxicity, pesticide resistance, cost, and availability [90].

Organic farming is a rapidly developing more sustainable method that improves soil quality [38,91,92], thereby lowering the risk of negative environmental impacts such as ecosystem degradation and global warming [93]. This form of agricultural production aims to utilize non-chemical disease prevention tools rather than other treatments [92]. For instance, ecofriendly farming systems rely on the use of different physical, cultural, and biological management techniques to suppress the spread of pathogenic microorganisms [94]. These methods, with only a few exceptions, do not pollute the environment [95]. Nevertheless, the activity spectrum of non-chemical farming strategies is extremely variable, and they are often less effective than their chemical analogues [96,97]. Therefore, successful disease control may require the implementation of multiple methods [98]. Yoon et al. [79] used soil-dwelling predatory mites and an essential oil mixture to control tomato spotted wilt virus and thrips. Likewise, Schmitt and Seddon [99] reported that the simultaneous use of microbiological control agents and plant extracts may have an additive or even a synergistic effect. Meanwhile, Baysal-Gurel et al. [48] showed that cover crops in combination with solarization significantly reduced *Rhizoctonia* root rot severity.

As another option, Integrated Pest Management (IPM) is a redesigned form of intensive agricultural systems, in which non-chemical methods, botanicals, or other curative treatments are combined with synthetic chemicals [35]. Just as with organic production, the goal is not to fully eliminate disease-causing organisms but to prevent them from becoming damaging or dominant in the cropping system. As a result, an integration of different preventive tactics (i.e., chemical, biological, physical, and genetic means with cropping practices) may be more effective than a single method [100,101]. A survey conducted by Kabir and Rainis [102] showed that vegetable farmers in Bangladesh used additional methods (e.g., biological control, soil amendments, pheromone traps, manual cleaning, soil solarization, and botanicals), while pure pesticides were only applied when there were no other options, or the pest infestation was high. In a study by Fielding et al. [103] eight medicinal plant extracts were applied successfully in combination with kresoxim-methyl for the inhibition of gray mold on apples. Shlevin et al. [104] analyzed independent studies to evaluate the efficacy of soil solarization with fumigants in controlling various soil-borne pathogens (e.g., *Fusarium* species; root-knot nematodes; and the group of *Sclerotium cepivorum*, *Verticillium* spp., *Pyrenochaeta* spp., *Rhizoctonia* spp., and *Pythium* spp.). Overall, their results showed that the combined technique improved management efficacy compared to soil solarization only. In contrast, Deguine et al. [101] reported that these integrated methods were rarely adopted by farmers, and, as a result, pesticides remained the cornerstone of crop protection around the world.

## 4. Aromatic Plants as Biocontrol Agents

There are approximately 17,500 aromatic plant species throughout the world, either cultivated or collected in the wild, that are greatly used for cosmetic, preservation, flavoring, therapeutic, and pharmacologic purposes [105,106,107,108]. These herbs are responsible for the synthesis of a multitude of secondary metabolites (e.g., flavonoids, phenol compounds, terpenes, and nitrogen-containing chemicals), which are formed from primary compounds [47,109,110]. Some of these metabolites are produced and stored during the normal growth of plants, whereas others accumulate only in response to stress conditions [111].

Due to their strong bioactivity, aromatic plants and their constituents may be applied as part of environmentally friendly management programs [64,112] to improve soil quality [113] and protect plants from pathogens and insects [114]. In a cropping system, aromatic plants and their bioactive metabolites can affect phytopathogens directly or indirectly by inducing systemic resistance and defense responses, repelling viral vectors, or inhibiting the growth of phytopathogens by changing the structure of soil microbial community [115,116,117,118,119]. Therefore, introduction of these noncrop biocontrol plants can affect crop productivity [120], with the postextraction residues and other plant debris being further sources of bioactive components that can be recycled as natural pesticides, organic amendments or transformed into value-added products providing nutrients, organic matter, and beneficial microorganisms [64,121,122]. For these reasons, the following sections provide background information on the applicability of aromatic and medicinal plants, botanicals, postextraction residues, and composts derived from aromatic plant waste to control the spread of major soil-borne pathogens.

### 4.1. Use of Pure Extracts as Biopesticides

Microorganisms are considered to be the most important biopesticides in the fight against plant diseases, whereas botanicals still represent a relatively small part of the market. Prior to World War II, only four major components were commonly used, i.e., rotenone and rotenoids, pyrethrum and pyrethrins, nicotine and alkaloids, and some vegetable oils [123], while other allelochemicals, such as derris and citronella oil, were applied as insecticides [90]. Although their use declined following the commercialization of synthetic agrochemicals due to their instability (pyrethrum) and toxicity to nontarget species (rotenone and nicotine) [123,124], the multitudinal backlash against synthetic compounds eventually led to the prosperity of biopesticides of plant origin [125].

The group of botanicals includes various plant volatiles, extracts, and natural oils that are characterized by improved biocompatibility, little or no cross-resistance to commercial chemical agents, and structural diversity [126,127]. Generally, these biologically active substances are extracted or distilled from fresh or dried plant materials with classical methods using water, alcohol, and other solvents [127]. To decrease the environmental impact, green processes requiring no or low amounts of solvents and less energy and time have also been developed including subcritical water extraction, pressurized ultrasound and microwave extraction, and supercritical fluid extraction [128,129,130].

#### 4.1.1. Essential Oils

Essential oils (EOs), biosynthesized, stored, and secreted in various parts of aromatic herbs, e.g., buds, bark, leaves, zest, twigs, wood, stems, seeds, fruits, rhizome, roots, and flowers [128,131,132,133], are complex mixtures of terpenoids and phenolic compounds that play a critical role in plant defense mechanisms against abiotic and biotic stress factors [128,132,134]. These volatile organic compounds can diffuse through water- and gas-filled pores and serve as food sources, infochemicals, chemo-attractants, or antimicrobials [135]. Monoterpenes, as frequent constituents of EOs, are involved in chemical and ecological interactions, and their production may be induced by the attack of pathogens and herbivores [136,137]. Due to their bioactive potential, EOs and their constituents can be utilized as components of ecological products with nematicidal, antimicrobial, antiviral, insecticidal, repellent, antifeedant, and molluscicidal activity [23,118,138]. They can also support the growth of other soil microbes indirectly by killing organisms and providing easily decomposable carbon and energy sources [139]. This was confirmed by [140], who found that *Mentha × piperita* EO had an instant antimicrobial effect on soil; however, a fraction of soil microbes survived the treatment and used the killed microorganisms as fresh organic carbon. As a result, an increased microbial biomass and respiration rate were recorded.

As for the efficiency of active substances derived from aromatic and medicinal plants, EOs tend to possess stronger antimicrobial properties than other extracts [51]. Although several studies investigated the biocidal effect of EOs and their constituents on phytopathogens [46,141,142], the mechanism of their action is complex and not fully understood [143,144]. Volatile oils containing oxygenated monoterpenes, primarily phenols and aldehydes, tend to be more active against pathogens [145,146]. This was confirmed by Wogiatzi et al. [147], who found that the thymol-rich EO of *Origanum vulgare* collected from the mountain Olympus in Greece had the strongest antifungal effect on *Verticillium dahliae, Pythium* spp., and *Sclerotinia sclerotiorum*. Conversely, the hydrocarbonic fraction of *Salvia pomifera* L. ssp. *calycina* (Sm.) Hayek was less effective against *Sclerotinia sclerotiorum* and *Rhizoctonia solani* than was the oxygenated fraction [148].

The application of EOs can provoke chemical, physical, and biochemical changes in the cell [149] including disruption of the permeability barrier and proton pump function, damage of cytoplasmic membrane, increased permeability and leakage of cell contents, decreased ATP synthesis and augmented ATP hydrolysis, cytoplasm coagulation, reduced membrane potential, and inhibition of the production of toxic microbial metabolites [133,150]. As regards fungi, EOs can inhibit ergosterol biosynthesis leading to the depletion of sterol content in the cell membrane, disruption of cell membrane integrity and permeability, loss of ions, and, eventually, inhibition of fungal growth [151]. The study of Upadhyay et al. [151] has shown that a decreased level of methylglyoxal may also be observed that was reported to be in correlation with aflatoxin B1 production in *Aspergillus flavus*. In addition, Vokou et al. [139] claimed that volatile oils extracted from *Satureja thymbra* could reduce the spore germination and mycelial growth of *Penicillium citrinum* and *Mucor hiemalis*, respectively. Another study conducted by Sempere-Ferre et al. [152] showed that EO constituents, either alone or in combination, inhibited the mycelial growth of *Botryotinia fuckeliana* and *Rhizoctonia solani*, although eugenol exerted a fungistatic effect only. Similarly, testing the antifungal efficacy of various EO constituents, Marei et al. [153] found that 1,8-cineole, (R)-camphor, (R)-carvone, camphene, cuminaldehyde, (R)-linalool, geraniol, (1R,2S,5R)-menthol, (S)-fenchone, myrcene, thymol, and (S)-limonene were promising substances against *Fusarium oxysporum*, *Penicillium digitatum*, *Aspergillus niger*, and *Rhizoctonia solani*. Thymol and (S)-limonene possessed strong inhibitory effect on cellulase and pectin methyl esterase activities. Lee et al. [154] stated that *Origanum vulgare* EO had a broad antifungal spectrum against both postharvest (*Botrytis cinerea*, *Colletotrichum gloeosporoides*) and soil-borne pathogenic fungi (*Fusarium oxysporum*, *Rhizoctonia solani*, and *Pythium ultimum*). Bi et al. [155] reported that a 21-day-long soil treatment with EOs from palmarosa (*Cymbopogon martini*), oregano (*Origanum syriacum*), and red thyme (*Thymus vulgaris*) at concentrations of ≥0.1 μg/mL decreased the population density of *Phytophthora capsici* below the limit of detection.

As nematicides, EOs can reduce gall formation, hinder the hatching of nematode eggs, immobilize juveniles, and, as a result, completely suppress nematode infestation [156]. This is consistent with the findings of Ntalli et al. [65], who reported the EOs of *Origanum vulgare*, *Origanum dictammus, Mentha pulegium*, and *Melissa officinalis* to be effective against *Meloidogyne incognita* by irreversibly paralyzing the larvae (Table 1). In fact, the oxygenated EO constituents were more active than hydrocarbons, including β-caryophyllene, *p*-cymene, and limonene.

Overall, the studies presented thus far provide evidence that the biological activity of EOs is usually strongly related to their chemical composition; the synergistic effect of different bioactive components [65,148]; specific seasonal, geographic, and climatic conditions [64]; genetic factors; and harvest and postharvest processes [157].

#### 4.1.2. Phenolic Compounds

Aromatic plants are rich sources of phenolic compounds (e.g., phenols, coumarins, tannins, flavonoids, phenolic acids) derived from the phenylpropanoid metabolism and shikimate pathway [158,159]. So far, around 8000 phenolics with highly diverse structures have been reported in the literature [160,161]. They contain one or more aromatic rings (C6) bearing at least one hydroxyl group [162,163]. These compounds are toxic to fungi, bacteria, insects, nematodes, and weeds [164], but, unlike EOs, they are water-soluble because they mostly exist in glycosidic form in plants [165].

Although polyphenols were isolated from various plant parts [166], they are found at higher levels in the outer layers of tissues [167] as the production of these metabolites can be induced by both biotic and abiotic factors such as reactive oxygen and nitrogen species, insufficient amounts of nutrients, low temperatures, UV light, predators, parasites, pathogens, wounding, and other stress conditions [163,168,169]. Nevertheless, the antimicrobial potential of polyphenols has not been fully deciphered [170] due to the complexity of their natural mixtures in plants [171] and their inconsistent activity [172]. Among them, flavonoids are associated with the stronger biocidal effects, whereas nonflavonoid compounds usually show a weak antagonistic behavior against pathogens [173]. Their activity, however, depends not only on their purity, structure, and concentration but also on the tested microbial strains, solvents, and experimental conditions [172].

Natural phenolics combine seemingly antagonistic effects, including an antioxidant activity, with variable degrees of cytotoxicity [174]. The mode of action of phenolics involves modification of cell membrane permeability and changes in intracellular functions, such as [170] inhibition of efflux pump and enzymatic (i.e., DNA gyrase, beta-ketoacyl acyl carrier protein synthase II and III, FabG, FabZ, FabI, d-alanine–d-alanine ligase, urease, sortase A, dihydrofolate reductase, etc.) activities, leakage of intracellular constituents, disturbance of cell wall biosynthesis, and inhibition of bacterial biofilm formation [175,176]. Important phenolics can repress or induce the expression of genes playing important roles in the pathogenesis of soft-rot-causing pathogenic bacteria or induce genes encoding efflux pumps in soft-rot enterobacteria [174]. As Summers and Felton [177] stated, phenolics could result in reduced protein digestibility, impaired enzymatic functions, and reduced bioavailability of amino acids by forming complexes with proteins. In addition, Simmonds [178] mentioned that the ingestion of phenolic compounds can reduce the nutritive value of the food consumed by nonadapted insects. This effect may be due to the induction of oxidative stress to the digestive system [177]. Widmer and Laurent [179] reported that lavender (*Lavandula angustifolia*), lavender hybrid (*Lavandula angustifolia* × *Lavandula spicata*), and rosemary (*Rosmarinus officinalis*) leaf extracts reduced the germination of *Phytophthora capsici*, *Phytophthora megakarya*, and *Phytophthora palmivora* zoospores. The aqueous extract of *Acacia saligna* H. L. Wendl. containing benzoic acid, caffeine, o-coumaric acid, naringenin, quercetin, and kaempferol effectively inhibited the mycelial growth of *Fusarium culmorum*, *Penicillium chrysogenum*, and *Rhizoctonia solani*, especially at high concentrations (Table 2). At the same time, the minimal inhibitory concentration of the extract was 200, 300, 300, and 100 µg/mL against *Agrobacterium tumefaciens*, *Enterobacter cloacae*, *Erwinia amylovora*, and *Pectobacterium carotovorum* subsp. *carotovorum*, respectively [180]. The filter-sterilized water extracts of *Origanum vulgare* L., *Melissa officinalis* L., and *Salvia officinalis* L. shoots (0.5 and/or 2.0% *w*/*v*) suppressed the propagation of *Fusarium oxysporum* f. sp. *asparagi* [181]. Similar findings were reported by Ahmad and Matsubara [182], who demonstrated that the aqueous extract of *Thymus vulgaris* L. (0.5% and 2.0%) had the maximum suppression effect on *Fusarium oxysporum* f. sp. *cyclaminis*. Caffeic acid and rosmarinic acid, as the main constituents, also exhibited a stronger antifungal activity. To induce the defense responses of soybean and sorghum, Colpas et al. [112] used the aqueous extracts of *Ocimum gratissimum* leaves, which caused an instant increase in phytoalexin production. López et al. [183] demonstrated that the dichlormethanolic extracts of *Melissa officinalis* L. subsp. *officinalis*, *Mentha longifolia* (L.) Hudson, *Origanum vulgare* L. subsp. *virens* Bonnier & Layens, and *Salvia pratensis* L., as well as the ethyl acetate extract of *Mentha × piperita* L., *Salvia pratensis* L., and *Thymus praecox* Opiz subsp. *Polytrichus* inhibited the growth of *Rhizopus stolonifer* over 20% under in vitro conditions. The nematicidal activity of aromatic plant extracts was demonstrated by Ntalli et al. [64]: *Origanum vulgare* L. and *Thymus citriodorus* (Schreb) extracts were effectively utilized against *Meloidogyne javanica* (Treub) and *Meloidogyne incognita* (Kofoid and White). However, elevated concentrations of the active constituents may have an inhibitory effect on physiological processes of crop plants by increasing electrolyte leakage from the cell membrane of seedlings and negatively affecting seed germination, chlorophyll synthesis, and the functioning of photosystem II [184].

#### 4.1.3. Applicability of Pure Metabolites in Plant Protection

Plant-derived biopesticides are inexpensive and readily available materials, which do not contaminate the environment due to their biodegradable nature [47,127]. Despite the extensive body of scientific literature and the tremendous amount of experience that support the bioactivity of botanicals on plant pathogens, only a few botanical pesticides are available and utilized in agriculture due to the lack of field verification [127,185]. In Hungary, for instance, the following plant-based pesticides and plant conditioners have been approved: Green Nanoherb^TM^ and Biomit^®^ with a blend of EOs, DU-OL^TM^ with lavender and lemon oil, ORGANIC^TM^ with orange oil, and Herbal^TM^ containing herbal extracts [186,187].

Although several plant-based protectants, e.g., spearmint oil and clove oil, have even been included in the pesticide database of the European Union and approved by the European Commission [149,188], the commercialization of botanical extracts has been limited by regulatory policies, costs, and prolonged procedures [47]. These bioactive substances can hinder the growth of soil-borne pathogens with a relatively mild effect on the nontarget species [35]. However, they are prone to decompose rapidly, whereas moisture, air, or sunlight may affect their stability, efficiency, and shelf-life. In addition, there are further drawbacks that can complicate the applicability of botanical extracts in the field, such as their nonselective nature, varying performance, and unavailability during certain parts of the growing season, as well as the absence of legislation; established residue tolerance; scientific validation; and sufficient data on stability, quality, and effect on human health [127]. The applicability of volatiles or other extracts may be also compromised by their dose-dependent properties [53,184].

Overall, plant-based bioactive substances may be integrated into common practices to reduce the use of conventional chemical treatments [127]. Formulation with emulsifying agents, polymers, surfactants, stabilizers, defoamers, solvents, and other substances can be used to ensure their stability, improve their efficiency, and control the release of biologically active components under field conditions [114].

### 4.2. Aromatic Plants as Part of Agricultural Practices: Cultivating Aromatic Plants and Recycling the Waste as Green Manure and Compost

**Figure 2:**
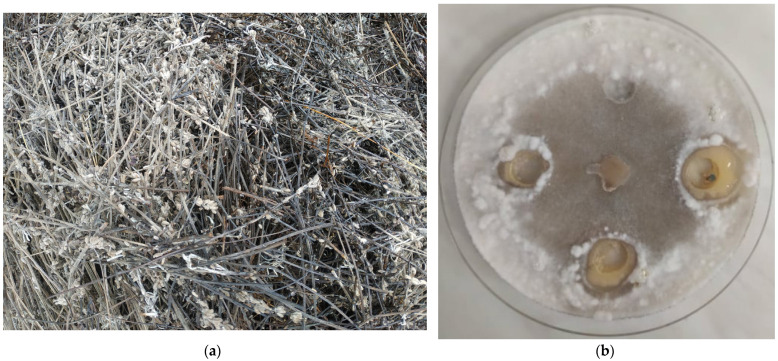
(**a**) Post-extraction lavender (*Lavandula angustifolia*) waste; (**b**) Antifungal effect of lavender waste extracts on phytopathogenic *Sclerotinia sclerotiorum*. Photographs taken by Babett Greff.

The aerial parts and roots of aromatic plants and their waste can affect the composition of microbial populations in soil by releasing complex mixtures of chemicals (Figure 2). These secondary metabolites, excreted directly into the soil from the living and decomposing tissues [109,189], may inhibit the growth of phytopathogens [190].

Over the last decades, various forms of these herbs including cultivated plants, green manure, compost, and wastes from EO extraction (plant residues and hydrosol) have been tested as potential non-chemical treatments against soil-borne plant pathogens. In the next subsections, the potential effects of aromatic plants, their postextraction wastes, and compost on phytopathogens will be discussed.

#### 4.2.1. Diversifying Cropping Systems with Aromatic and Medicinal Plants

As continuous cropping systems have been constrained by various biotic and abiotic factors (i.e., reduced soil quality, autotoxicity of the crop, shifts in microbial communities, reduced microbial diversity and abundance, prosperity of plant pathogens, etc.) [19], agricultural diversity has become a critical part of sustainable agroecosystems [191]. Diversification requires the combination of environmental and economic sustainability through increasing crop species diversity (intercropping, crop rotation) or planting noncrops (cover-cropping) [19,191,192,193]. These strategies may reduce production costs and improve resource use and soil quality [192], while the highly diverse plant communities thus created are less affected by phytopathogens [193]. However, the reduced activity of plant pathogens in such communities depends both on host density and the effects of neighboring species on disease-causing organisms [43].

Under optimal conditions, the phytomanagement of soils through the cultivation of aromatic and medicinal plant species can be a win-win approach that provides additional profits and marketable products [194,195,196]. As a vegetation cover, herbs can increase the fertility and the organic matter content of soils resulting in improved properties (e.g., water retention, soil porosity, permeability, and bulk density) [105].

It is also believed that the microbiota around medicinal and aromatic plants is highly plant-specific [196]. The rhizospheric zone in the vicinity of the plant roots is an attractive environment for a large number of organisms because plants may release up to 20% of the photosynthetically fixed carbon through their roots [119]. In addition to the typical root exudates, such as sugars, organic acids, amino acids, and sterols, aromatic crops can release novel chemicals in the soil that may have a long-lasting effect on the processes arbitrated by the surrounding microorganisms. Although the production of plant secondary metabolites is relatively low [110,116,197], volatiles can form a stable concentration gradient in the soil due to the lack of turbulence [109], and the build-up of pathogenic microorganisms and other predators can thus be inhibited [191]. However, several rhizospheric microorganisms have the potential to adapt and conduct endophytic or epiphytic life. These microbes and the host plant will influence each other through biological and physicochemical interactions [198]. For instance, the microorganisms associated with the roots of aromatic plants take part in plant growth and nutrition, regulate disease interactions [196], and may induce the production of certain bioactive compounds [117].

Over the past decades, several studies have confirmed the beneficial effects of aromatic herbs on soil microbial composition. Karthikeyan et al. [199] showed that the bacterial, fungal, and actinomycete populations in the rhizosphere of *Ocimum sanctum* L. were 2.3 × 10^7^, 1.9 × 10^5^, and 1.2 × 10^6^ CFU/g, respectively. These values were higher than the corresponding ones observed in the rhizosphere of *Coleus forskholii* Briq. Similar results were reported for diazotrophic bacterial populations (i.e., *Azospirillum* spp., *Azotobacter* spp., and *Pseudomonas* spp.). Examining soil microbial populations, Adamović et al. [200] observed an increase in the total number of microorganisms, cellulolytic microorganisms, azotobacters, fungi, and free nitrogen-fixing microorganisms when mint (*Mentha × piperita* L.) was planted. The cultivation of basil (*Ocimum basilicum* L.) had the same effect on the total numbers of actinomycetes. Zhang et al. [113] showed that intercropping pear trees with basil or summer savory (*Satureja hortensis* L.) could increase the organic matter content of soil and the activities of enzymes including invertase, urease, and catalase due to the influence of root exudates on soil microbial diversity. Meanwhile, soil organic matter was negatively correlated with pathotrophic groups of fungi, and, thus, intercropping significantly reduced the relative abundance of pathotrophic and saprotrophic groups. Bais et al. [111] reported that the fungal cell wall elicitors from *Phytophthora drechsleri* and *Phytophthora cinnamomi* improved root growth of *Ocimum basilicum* L. and facilitated rosmarinic acid production in hairy root cultures. Former studies have demonstrated that both EOs and aromatic water may possess antiphytoviral activity [80], but the physicochemical properties of soil and root exudates can also influence the migration and location of these organisms [74]. Due to their phytoremediation potential, several aromatic and medicinal plants are also used for the remediation of heavily contaminated soil by accumulating and removing organic and inorganic contaminants [201].

It should be noted that consecutive monocultures of herbs may severely alter the bacterial and fungal populations in soil [202]. According to the research conducted by Tang et al. [19], the continuous cropping of *Salvia miltiorrhiza* Bunge affected the microbial composition (e.g., fungal and actinomycete communities) of soil both in terms of structure diversity and abundance. Overall, the productivity of these plants tends to decrease over time because of the accumulation of their natural enemies [43] and the relatively low diversity of functional microbes [19].

Crop rotation is one of the best options to keep the levels of soil-borne pathogens and weeds low, but, from an economical perspective, it is not always acceptable [44,203]. Cover cropping and intercropping are used more and more frequently in conventional habitat management systems [138]. Aromatic herbs are excellent intercropping options for different cultures due to their characteristic traits and resistance to adverse environmental conditions [204]. Verma et al. [192] found that intercropping *Pelargonium graveolens* L. with companion crops may improve the postharvest soil total Kjeldahl nitrogen; organic carbon; carbon-to-nitrogen ratio; and available N, P, and K contents. The cultivation of wheat, oat, and barley also enhanced the biomass-specific respiration of soil that might be in association with higher microbial activity. Khan et al. [194] reported that cropping treatments with *Ocimum basilicum* L. cv. CIM-Saumya and *Mentha arvensis* L. cv. Kosi along with crop residue retention showed reduced CO_2_-C emission and increased soil organic carbon content compared to fallow soil. Chand et al. [205] showed that the cultivation of *Pelargonium graveolens*, *Rosmarinus officinalis*, and *Mentha piperita* had a conservation function, and these herbs reduced soil erosion and slowed down the runoff. However, the relative soil and water conservation efficiency of *Thymus vulgaris* was almost zero due to its poor canopy coverage. The authors also suggested that geranium and rosemary were good candidates for vegetative barriers, intercrops, and cover crops.

The plant material of herbs can be left on the top of the soil as mulch or incorporated into green manure [206]. In this form, they can interfere with phytopathogens directly through the release of toxic compounds or indirectly [207] by favoring the natural enemies of soil-borne pathogens through habitat manipulation [35]. Furthermore, the supplementation of plant growth-promoting microorganisms associated with medicinal plants can improve plant health by increasing their immunity to phytopathogens [208], improving stress tolerance, and influencing nutrient availability and uptake, as well as the production of growth-promoting metabolites and hormones, such as cytokinins and auxins [209]. Castronovo et al. [210] reported that microorganisms isolated from the bulk soil of *Origanum vulgare* L. exhibited antibacterial activity against various human pathogens. The *Agrobacterium*, *Agromyces*, *Bacillus*, and *Chryseobacterium* isolates inhibited the growth of several *Bacillus* strains of environmental origin. Tiwari et al. [211] isolated potential plant growth-promoting bacterial agents (*Bacillus* and *Pseudomonas* spp.) from the root vicinity of medicinal aromatic plants such as *Ocimum* spp. Under greenhouse conditions, the isolated bioinoculants alone or in combination with *Trichoderma harzianum* reduced the reproduction factor of *Meloidogyne incognita* by 46.4–72.3% in sterile and natural soils. Chowdhary and Kaushik [212] reported that approximately 12% of endophytic fungal isolates from *Mentha* × *piperita* plants exhibited antifungal activity against three or more of the tested phytopathogens.

In addition to the aforementioned beneficial effects on soil microbial composition, aromatic plant-based cropping systems can provide significantly increased profits and crop production rates [213] because herbs (e.g., *Ocimum basilicum* L.) are less prone to compete with cultivated crops for nutrients [214,215]. Carvalho et al. [216] studied how intercropping with aromatic plants, such as *Ruta graveolens*, can affect tomato yield. This cultivation technique was found to increase the total yield of tomato fruits by 26%. In a similar companion planting experiment, Ahmad et al. [214] observed that certain Lamiaceae species (e.g., *Ocimum basilicum* L., *Mentha piperita* L., *Hyssopus officinalis* L.) promoted the growth of tomato plants and influenced the production and accumulation of certain metabolites in the dominant crop. Peppermint and hyssop enhanced shikimic acid and apigenin contents in all parts of tomato plants providing a product with improved quality and yield. Likewise, cropping with basil resulted in higher free amino acid contents with the leaves being rich in alanine, phenylalanine, iso-leucine, lysine, valine, and γ-aminobutyric acid (GABA), whereas the stems contained elevated levels of valine, serine, alanine, proline, GABA, and glutamine compared to the control treatment. In addition, the 1:1 tomato–aromatic plant companion setups boosted the growth of tomato plants without the accumulation of competition pressure. Under certain circumstances, however, temporal phytotoxic effects could be experienced due to the incorporation of aromatic herb biomass. Nevertheless, other techniques (e.g., relay-planting) are available that can optimize productivity [217].

#### 4.2.2. Recycling the By-Products of Aromatic Plant-Utilizing Sectors

The demand for aromatic and medicinal plant-based products has increased continuously, at the rate of 15–25%, for the last few years [218]. As the ratio between the processed EO and the utilized plant material is low [219], large quantities of solid waste are generated and remain unutilized [220]. From a sustainability perspective, the aromatic biomass cannot be considered as waste [221], and it may be recycled as animal feed, biosorbents, biogas, biofuel, chelating agents, biopesticides, or soil amendments [121,219].

Just as with the fresh plant material, the distilled solid biomass can contain volatile components and phenolic compounds, albeit in different ratios [219,222]. According to Kadoglidou et al. [33], the application of *Mentha spicata* L. and *Origanum vulgare* L. ssp. *hirtum* plant material as soil amendments (4%) decreased the average degree of infection (*Fusarium oxysporun* f. sp. *lycopersici* and *Verticillium dahliae*) of tomatoes. In addition to plant disease reduction, the growth and physiology indices as well as soluble solids contents and fruit yield were also affected. Moreover, the amendment of *Mentha spicata* L. tissue at different concentrations may have a positive impact on tomato seedling emergence and dry weight, the size of the most robust leaf, shoot length, physiological parameters (e.g., stomatal conductance, photosynthetic rate, and yield), and the size of fungal and bacterial populations [223]. Klein et al. [224] used herbal biomass in combination with soil solarization to control the spread of *Fusarium oxysporum* f. sp. *radicis-lycopersici* and *Meloidogyne javanica* under farm conditions. The results showed that solarization with rosemary (*Rosmarinus officinalis* L.), sage (*Salvia officinalis* L.), tarragon (*Artemisia dracunculus* L.), and thyme (*Thymus vulgaris* L.) residues was highly effective against the tested fungi reducing their viability by 99–100%. As for the root-knot nematode, galling was reduced to zero in tomato roots in the solarized soil amended with sage and thyme residues. Despite the positive effect of untreated herbal residues on soil productivity and disease severity, their direct use may be compromised due to their unknown composition [157]. Furthermore, the introduction of novel plant metabolites may also affect indigenous microbial communities [116], critically the decomposition process [225], and endanger crop growth and yield [203]. As an example of the negative impact, Gravanis et al. [226] reported that the incorporation of dried *Origanum vulgare* biomass significantly decreased the number of bacterial colonies in soil samples. Similar results were reported by Chouliaras et al. [227]. In contrast, Ainalidou et al. [228] showed that soil enrichment with the green parts of *Mentha spicata*, *Mentha piperita*, and *Rosmarinus officinalis* increased the total microbial biomass. However, the use of rosemary had a negative effect on the growth indices of tomato seedlings, including weight, shoot length, and root length of fresh seedlings, as also reported by Argyropoulou et al. [229].

The meaningful utilization of aromatic biomass is still a great challenge. Composting and vermitechnology are commonly applied to treat these agricultural wastes prior to use. The mature and stable end product is a suitable soil amendment that enhances important soil processes and features improving the diversity and activity of microbial communities in soil [223,230]. Moreover, composts provide a new environmental source of microbes with antimicrobial activity [231]. Although their efficiency is variable, the microorganisms involved in the decomposition of organic matter can suppress the growth of soil-borne pathogenic microbes through antagonistic interactions, inducing systemic resistance in the host plant or forming humic molecules and biostimulants [232]. Under glasshouse conditions, the nematicide activity of vermicomposts containing *Artemisia annua*, *Chrysanthemum cinerariaefolium*, *Plantago ovata*, *Mentha arvensis*, *Pelargonium graveolens*, and *Tagetes minuta* was examined by Pandey and Kalra [233]. The maximum reduction in root galling was recorded in tomato plants treated with menthol mint vermicompost [Root-Knot Index (RKI): 1.33] followed by marigold (RKI: 1.66), isabgol (RKI: 1.66), and qinghao (RKI: 1.81). Compared to one of the most common chemical nematicides (carbofuran), increased fresh and dry root and shoot weights and fruit yields were achieved with certain herb-based vermicompost treatments. Singh et al. [234] prepared vermicompost from distillation waste of aromatic oil crops (e.g., *Cymbopogon flexuosus* and *Cymbopogon winterianus*) with *Eisenia fetida* to control root-rot of *Coleus forskohlii* caused by *Fusarium chlamydosporum* and *Ralstonia solanacearum*. Zhou et al. [235] co-composted food waste and sawdust with Chinese medicinal herb residues (1:1:1). The acetone extract of the end products showed antagonistic activity against *Fusarium oxysporum* and *Alternaria solani*, but the antimicrobial properties of the mature compost were as strong as those of the Chinese herbal residues only. It was concluded that the growth inhibition observed was partly due to the natural microbiota of the compost. In eleven composts, Zaccardelli et al. [122] found 104 spore-forming bacterial isolates that exerted antagonistic effects on *Sclerotinia minor* and *Rhizoctonia solani*. Among the seven most promising isolates, the in vivo antipathogenic activity of two *Bacillus subtilis* strains and a *Bacillus amyloliquefaciens* isolate was confirmed against *Sclerotinia minor* on *Diplotaxis tenuifolia* L.

In addition to the solid by-products, the hydrolates remaining after the extraction process may also possess biocidal properties [236] as a result of leaching of certain bioactive compounds (i.e., trace amounts of EOs and other water-soluble components) during hydrodistillation [219,237]. The hydrosol extract of *Thymus capitatus* L. rich in carvacrol effectively inhibited the growth of important fungal pathogens of *Citrus sinensis* L. including *Aspergillus niger*, *Aspergillus oryzae*, and *Fusarium solani* [238]. Gaspar-Pintiliescu et al. [239] reported antibacterial activity for the aromatic water of *Rosmarinus officinalis*. Under in vitro conditions, strong nematicidal activity was associated with the use of hydrolates derived from *Lavandula* × *intermedia* Emeric ex Loisel. var. super and *Lavandula luisieri* (Rozeira) Rivas-Martínez [240]. A similar effect of *Thymus citriodorus* (Schreb) was demonstrated by Ntalli et al. [241] against *Meloidogyne incognita* and *Meloidogyne javanica*. Sainz et al. [242] reported a high mortality rate for second-stage juveniles of *Meloidogyne javanica* upon use of a hydrolate from *Artemisia pedemontana* subsp. *assoana*. However, hydrolate-based natural pesticides may cause acute toxicity at higher doses for non-target organisms, reduce the metabolism of the natural soil microbiota, and slightly influence the physiological diversity of microbial communities [243].

## 5. Conclusions

Modern agriculture still relies heavily on the application of synthetic chemicals, even though controversies have arisen regarding their safety. Due to the public’s concerns, the strict regulations, and the pursuit of agricultural sustainability, low-risk and environmentally compatible biocontrol alternatives have emerged as suitable substitutes for these agrochemicals. Botanicals, including EOs and phenolic compounds, have been used for decades as non-chemical treatments thanks to their biodegradable nature and high selectivity. Their market is continuously growing; however, they only make up a small percentage of the global market, and just a few of these aromatic plant-based biopesticides are commercially available because of their varying performance and the absence of field studies regarding their efficiency. The inclusion of aromatic and medicinal herbs into agricultural practices is a possible way to increase crop diversity. In addition, the use of their industrial by-products as organic fertilizers may suppress the growth of major soil-borne phytopathogens. The cultivation of aromatic herbs can also improve crop production rates and the chemical composition of other plants. If used as part of integrated pest management programs, they are capable of reducing the environmental impacts of chemical pesticides. In the last few decades, disposal of by-products (e.g., solid biomass, aromatic water, etc.) generated by the aromatic and medicinal plant-utilizing sector has become a common issue throughout the world. Since these wastes may be rich sources of bioactive compounds and beneficial microorganisms, their use as natural biopesticides or organic amendments has also gained popularity. Composting and vermicomposting technologies can offer a meaningful way to produce a mature and stable end product that can improve important soil processes and provide nutrients for the treated crops.

All in all, botanicals, and especially EOs, are the most studied and commonly used treatments against soil-borne plant pathogens. There is substantial evidence that several of them possess strong antifungal properties, thereby reducing mycelial growth and/or spore germination of various fungal taxa such as *Fusarium*, *Aspergillus*, *Penicillium*, *Rhizoctonia solani*, *Verticillium dahliae*, and *Sclerotinia sclerotiorum*. Moreover, they show pronounced nematicidal activities against phytoparasitic, root-knot nematodes (*Meloidogyne* spp.). Nevertheless, the application of these methods is far behind their true potential due to some shortcomings, e.g., lack of in vitro experiments, varying selectivity, diversity of bioactive compounds and complex mechanisms of action, rigorous approval procedures and registration processes, etc. Therefore, more insight is required to overcome these obstacles and improve the overall commercialization process of plant-based biocontrol agents.

## Figures and Tables

**Table 1:** Properties of essential oils and/or essential oil constituents, dealt with in this review paper, against soil-borne organisms and related references.

Essential Oil (EO)	Target Organism	Effect	Reference
*Thymus pallescens*, *Artemisia herba*-*alba* Asso, *Laurus nobilis* L., *Cymbopogon citratus* (de Candolle ex Nees) Stapf.	*Fusarium oxysporum* f. sp. *ciceris* Padwick	Inhibition of mycelial growth, sporulation, and spore germination	[46]
*Mentha piperita* L., *Thymus vulgaris* L., *Lavandula angustifolia* Mill.	*Verticillium dahliae* Kleb. isolates	Inhibition of mycelial growth	[51]
*Origanum dictamnus*, *Mentha pulegium*, *Origanum vulgare*, *Melissa officinalis*	*Meloidogyne incognita*	High nematicidal activity (irreversible motility loss of nematode juveniles)	[65]
*Satureja thymbra*, *Rosmarinus officinalis*	*Penicillium citrinum*	Suppression of spore germination	[139]
*Satureja thymbra*	*Mucor hiemalis*	Complete inhibition of mycelial growth	[139]
*Mentha* spp.	*Penicillium expansum*	Inhibition of mycelial growth	[141]
*Origanum onites* L., *Salvia thymbra* L.	*Alternaria alternata*, *Aspergillus* spp., *Penicillium* spp., *Trichoderma viride*, *Cladosporium cladosporioides*, *Phomopsis helianthi*	Strong antifungal activity against the microorganisms tested	[142]
*Origanum* spp.	*Verticillium dahliae*, *Fusarium oxysporum* f. sp. *lycopersici*, *Sclerotinia sclerotiorum*, *Pythium* spp.	Inhibition of mycelial growth	[147]
*Salvia pomifera* L. ssp. *calycina* (Sm.) Hayek	*Fusarium oxysporum* f. sp. *dianthi*, *Fusarium solani* f. sp. *cucurbitae*, *Fusarium proliferatum*, *Verticillium dahliae*, *Sclerotinia sclerotiorum*, *Rhizoctonia solani*	Inhibition of mycelial growth	[148]
EO constituents (carvacrol, cinnamaldehyde, eugenol, thymol)	*Rhizoctonia solani*	Inhibition of mycelial growth	[152]
EO constituents [camphene, (R)-camphor, (R)-carvone, 1,8-cineole, cuminaldehyde, (S)-fenchone, geraniol, (R)-linalool, (1R,2S,5R)-menthol, myrcene]	*Aspergillus niger*, *Fusarium oxysporum*, *Penicillium digitatum*, *Rhizoctonia solani*	Inhibition of mycelial growth	[153]
EO constituents [thymol, (S)-limonene]		Inhibition of mycelial growth, inhibitory effect on pectin methyl esterase and cellulase	
*Cuminum cyminum*, *Eucalyptus citriodora*, *Origanum vulgare*	*Fusarium oxysporum*, *Pythium ultimum*, *Rhizoctonia solani*	Inhibition of mycelial growth	[154]
*Origanum syraicum*, *Cymbopogon martini*, *Thymus vulgaris*	*Phytophthora capsici* isolates	Strong antifungal activity, control of production and germination of sporangia and zoospores, oospore production, and mycelial growth reduction of *Phytophthora capsici* population in soil, suppression of infection on zucchini fruits	[155]

**Table 2:** Properties of extracts from aromatic and medicinal plants, dealt with in this review paper, against soil-borne organisms and related references.

Plant Extract	Type of the Extract	Target Organism	Effect	Reference
*Mentha piperita* L., *Thymus vulgaris* L., *Lavandula angustifolia* Mill.	-	*Verticillium dahliae* Kleb. isolates	Inhibition of mycelial growth	[51]
*Orginum vulgare*	Hydrosol from EO distillation	*Meloidogyne javanica*	Nematostatic/nematicidal activity (paralysis of second-stage juveniles)	[64]
*Orginum vulgare*, *Thymus citriodorus*	Water extract of plant powders	*Meloidogyne incognita*, *Meloidogyne javanica*		
*Rosmarinus officinalis*, *Lavandula angustifolia*, *Lavandula angustifolia × Lavandula spicata*, *Salvia officinalis*	Hot water extract of leaves	*Phytophthora* spp. isolates	Control of zoospore germination	[179]
*Acacia saligna*	Water extract of flowers	*Fusarium culmorum*, *Rhizoctonia solani*, *Penicillium chrysogenum*	Inhibition of mycelial growth	[180]
*Acacia saligna*	Water extract of flowers	*Agrobacterium tumefaciens*, *Pectobacterium carotovorum* subsp. *carotovorum*	Inhibition of bacterial growth	[180]
*Origanum vulgare* L., *Salvia officinalis* L., *Melissa officinalis* L.	Water extract of shoots	*Fusarium oxysporum* f. sp. *asparagi*	Suppression of fungal propagation	[181]
*Salvia officinalis* L.	Water extract of shoots	*Fusarium oxysporum* f. sp. *cyclaminis*	Suppression of fungal pathogen, reduction of disease indices in cyclamen roots and shoots	[182]

## Data Availability

Not applicable.
